# Local Anæsthesia by Nitrous Oxide: A Convenient Method of Applying It

**Published:** 1890-07

**Authors:** G. L. Curtis

**Affiliations:** Syracuse, N. Y.


### LOCAL ANÆSTHESIA BY NITROUS OXIDE: A CONVENIENT METHOD OF APPLYING IT.[1]

BY G. L. CURTIS, M.D., D.D.S., SYRACUSE, N. Y.

It is not surprising that the average man or woman, being aware of the highly sensitive organization of the teeth, should dread to submit to the dental operations frequently necessary for the preservation of those organs. The trifacial, from which the teeth draw their nervous supply, has been demonstrated to be the most sensitive nerve of the entire system. There is, perhaps, no more pressing need in dentistry than a safe, reliable means for rendering dental operations painless. What is wanted is an obtundent or local anæsthetic whose effect, while certain and complete so far as its action upon the part to which it is applied is concerned, may be exhibited without inconvenience, or producing unconsciousness of the patient, and without liability of causing subsequent injurious results. Such an agent, by abolishing the pain of dental operations, would lessen

---

[1] Read before the New York Odontological Society, March 18, 1890.

in a marked degree the average time consumed in performing them, and be a boon to patient and dentist.

The problem has been the subject of much thought among practitioners, and many methods have been proposed to achieve the end desired; but so far the results have been only partially or varyingly successful. The recognized facts that moisture is essential to sensation and that a tissue deprived of its moisture is dead to sensation points the way to the solution, and some of the methods which have been suggested were based on this idea. But the agents offered for the purpose have all been objectionable for one reason or another. Some failed to produce complete insensibility, or were uncertain in their action; some others exercised a destructive influence upon the tissues to which they were applied; while the exhibition of still others was attended with so much suffering to the patient as practically to bar their use.

This uncertainty in the action of drugs is well known, and is due in some instances to idiosyncrasies upon the part of the patient, at other times to a pathological condition of the tissues which prevents the action of the remedy, or to a lack of affinity between the drug and the tissue or organ to be treated.

Some time ago it occurred to me that a blast of nitrous oxide, under high pressure, thrown upon a tissue might have the effect of producing local anæsthesia by depriving the tissues of moisture and thus rendering them insensible to pain. The pressure of the gas in the cylinders, in which it is supplied in the liquefied form, is at ordinary temperatures about one thousand pounds to the square inch; that is, when the cylinder is full. This is ample for the purpose. I have here attached to a cylinder an apparatus which I have devised for the application of the gas, consisting of a flexible tube of sufficient strength to withstand the enormous pressure. The gas is forced, on opening the valve, through detachable tubes of various shapes and conveyed to the cavity, where it is applied by means of an automatic atomizer.

After experimenting upon myself for awhile, I became fully convinced of the efficacy of the device and the value of the idea. I then began to use it upon others as the opportunity offered, and was delighted to find that previous observations of its effects were confirmed. Exhibited as indicated, I have employed nitrous oxide as a local anæsthetic many times in my practice, with the most gratifying results in every case except two, in one of which the patient was hysterical, and the other in such a highly-wrought state of nervous excitability that I was unable to apply the blast properly. In order to show the difference in preparation of a cavity without and with the gas let me cite a typical case: Patient, male, aged forty-five years, had been for many years a great sufferer from nervous dyspepsia, complicated with functional derangement of the heart. At times his life was despaired of through heart failure. Without the aid of nitrous oxide, when I was excavating a cavity in one of his teeth, owing to the extreme sensitiveness of the dentine, his overtaxed and excitable nervous system gave way, his heart stopped beating, he became black in the face, and writhed in convulsions. It was only by the most active efforts that the circulation was restored and the patient revived. A temporary filling was inserted and patient dismissed.

He again presented upon a subsequent occasion with a superior central incisor, one-third of the crown of which had been lost by decay. The pulp was living, but with only a thin film of disintegrated dentine over it. On attempting to excavate, the pain produced was so excruciating that the patient, rising from the chair, declared that he would lose the tooth sooner than endure the torture. Thinking this an excellent opportunity to test the efficacy of the nitrous oxide as an obtundent, I endeavored to persuade him to allow me to try it. At length he reluctantly consented, but it was evident from the rigidity of his muscles, as he braced himself in the chair, and the frightened expression of his face that he had no faith in the success of the experiment. The blast was applied, and in three seconds the constrained look of the face gave way to a peaceful smile, and the patient exclaimed that he was free from pain. Then came a general abatement of the nervous symptoms, the muscles lost their rigidity, and the patient assumed a quiet, restful position. After applying the blast ten seconds, I began the task of removing the decay and preparing the tooth for filling, working as fast as the engine would operate the bur. The pulp was fully exposed, and a small drop of blood exuded, but the hemorrhage was checked without the aid of a styptic. In two minutes the cavity was ready. The pulp was capped and the entire filling completed without the slightest expression of discomfort from the patient, except when the gold was being finished at the neck of the tooth a half-hour later, which indicated the return of the pulp to its normal condition.

This tooth was carefully watched, and more than a year after the operation it was still in a perfectly healthy condition, and the first evidence of discomfort from it was yet to be felt. I have since treated several other teeth for the same gentleman by this method, and in each instance with equally satisfactory results.

A very similar case was that of a physician suffering from extreme nervous exhaustion, for whom I operated, under like conditions, in the same manner, except that it was not necessary to expose the pulp of the tooth. The operation was absolutely painless, and a year and a half afterwards the tooth had not manifested the slightest pain or discomfort.

Many other cases have occurred in my practice where the nitrous oxide blast was equally effective in obtunding the sensitiveness of the dentine. In fact, it has never failed of complete satisfaction except in the two instances before referred to, and these were so exceptional in their conditions that I feel that they should not be permitted to militate against the value of the treatment.

My experience is that sensation is perfectly restored to the obtunded tissue in from three to ten minutes after the application, in which respect the local action is very similar to that which follows the administration of nitrous oxide as a general anæsthetic. The duration, as also the degree, of the loss of sensation depends upon the length of the exposure to the blast. So far I have never observed any untoward after-effects which could be attributed to the exhibition of the nitrous oxide. It appears to be entirely innocuous, after the temporary effect passes off.

Considering the comfort to the patient, the ease and satisfaction to the dentist when operating upon the most sensitive tissue, and the rapidity of work thus rendered possible, as promised by the method described, it would seem to hold forth the hope of painless dentistry to thousands of sufferers who never visit the dental office, through fear of the torture they expect to undergo.

The advantages which I claim for this method, of producing local anæsthesia by nitrous oxide under high pressure, may be briefly summarized as follows:

The action is complete and certain. The tissues are to a greater or less extent deprived of moisture and perfectly obtunded.

Unless the application is pushed beyond all reason (which might result in the freezing of the parts) no injurious effect is produced upon the tissue, as when ether, alcohol, chloroform, carbolic acid, etc., are employed.

The application is not followed by inflammation. The patient is not rendered unconscious by it, and there is no unpleasant odor or soiling of the tissues, so objectionable in the use of the other agents referred to.

The parts being perfectly dry facilitates the most careful examination.

The agent (compressed or liquefied nitrous oxide) is always available in the cylinders supplied to dentists.

Neither the nitrous oxide nor its action is affected by the temperature of the surrounding atmosphere, nor is it combustible or explosive.

Just how far the advantages of the method can be applied in the major operations of surgery, as the removal of tumors, amputations, etc., is not yet determined. It is among the possibilities that the blast may be continuously applied in advance of the knife, so as to render the parts insensible, and at the same time reduce the loss of blood to the minimum. Certainly the lancing of boils or carbuncles may be accomplished by its aid easily and painlessly.

Whether the action of nitrous oxide, as herein set forth, is chemical or mechanical I am as yet unable to state, but by experiments, now in progress, I hope to arrive at a clear solution of the question. Thus far I am led to the belief that anæsthesia is the result of dehydration.

If the experience of others bears out the writer's testimony, it would seem reasonable to expect the general adoption of the method in dentistry and minor surgery. When so harmless an obtundent can so readily be applied, it would be unwarranted cruelty to inflict pain.

There are other fields in which this application of nitrous oxide to tissues promises untold usefulness. Thus, in microscopic technique, the preparation of tissues for making sections for examination formerly required months, whereas now it is accomplished in a few weeks by using alcohol for the extraction of the moisture, the length of time varying with the density of the tissue to be acted upon. But even this, as will be easily understood, is frequently unsatisfactory, as often an immediate examination of a suspected tissue is desirable so that its character may be determined promptly. Ether spray from a hand-atomizer has been used to some extent for quick work, but it is far from answering the requirements, and its use has been practically abandoned in some laboratories. It may be found that the nitrous oxide blast will be useful in this direction.

